# MEG and EEG data analysis with MNE-Python

**DOI:** 10.3389/fnins.2013.00267

**Published:** 2013-12-26

**Authors:** Alexandre Gramfort, Martin Luessi, Eric Larson, Denis A. Engemann, Daniel Strohmeier, Christian Brodbeck, Roman Goj, Mainak Jas, Teon Brooks, Lauri Parkkonen, Matti Hämäläinen

**Affiliations:** ^1^Institut Mines-Telecom, Telecom ParisTech, CNRS LTCIParis, France; ^2^Athinoula A. Martinos Center for Biomedical Imaging, Massachusetts General Hospital, and Harvard Medical SchoolCharlestown MA, USA; ^3^NeuroSpin, CEA SaclayGif-sur-Yvette, France; ^4^Institute for Learning and Brain Sciences, University of WashingtonSeattle WA, USA; ^5^Institute of Neuroscience and Medicine - Cognitive Neuroscience (INM-3)Forschungszentrum Juelich, Germany; ^6^Brain Imaging Lab, Department of Psychiatry, University HospitalCologne, Germany; ^7^Institute of Biomedical Engineering and Informatics, Ilmenau University of TechnologyIlmenau, Germany; ^8^Department of Psychology, New York UniversityNew York, NY, USA; ^9^Psychological Imaging Laboratory, Psychology, School of Natural Sciences, University of StirlingStirling, UK; ^10^Department of Biomedical Engineering and Computational Science, Aalto University School of ScienceEspoo, Finland; ^11^Brain Research Unit, O.V. Lounasmaa Laboratory, Aalto University School of ScienceEspoo, Finland

**Keywords:** electroencephalography (EEG), magnetoencephalography (MEG), neuroimaging, software, python, open-source

## Abstract

Magnetoencephalography and electroencephalography (M/EEG) measure the weak electromagnetic signals generated by neuronal activity in the brain. Using these signals to characterize and locate neural activation in the brain is a challenge that requires expertise in physics, signal processing, statistics, and numerical methods. As part of the MNE software suite, MNE-Python is an open-source software package that addresses this challenge by providing state-of-the-art algorithms implemented in Python that cover multiple methods of data preprocessing, source localization, statistical analysis, and estimation of functional connectivity between distributed brain regions. All algorithms and utility functions are implemented in a consistent manner with well-documented interfaces, enabling users to create M/EEG data analysis pipelines by writing Python scripts. Moreover, MNE-Python is tightly integrated with the core Python libraries for scientific comptutation (NumPy, SciPy) and visualization (matplotlib and Mayavi), as well as the greater neuroimaging ecosystem in Python via the Nibabel package. The code is provided under the new BSD license allowing code reuse, even in commercial products. Although MNE-Python has only been under heavy development for a couple of years, it has rapidly evolved with expanded analysis capabilities and pedagogical tutorials because multiple labs have collaborated during code development to help share best practices. MNE-Python also gives easy access to preprocessed datasets, helping users to get started quickly and facilitating reproducibility of methods by other researchers. Full documentation, including dozens of examples, is available at http://martinos.org/mne.

## 1. Introduction

Magnetoencephalography (MEG) and electroencephalography (EEG) measure non-invasively the weak electromagnetic signals induced by neural currents. While the more common neuroimaging method of functional magnetic resonance imaging (fMRI) provides volumetric images defined over voxel grids using a sampling rate of around one image per second, M/EEG captures both slowly and rapidly changing dynamics of brain activations at a millisecond time resolution. This enables the investigation of neuronal activity over a wide range of frequencies that can offer potentially complementary insights regarding how the brain works as a large system (Tallon-Baudry et al., 1997; Fries, 2009).

The processing and interpretation of M/EEG signals is, however, challenging. While fMRI provides unambiguous localization of the measured blood-oxygen-level dependent signal, estimating the neural currents underlying M/EEG is difficult. This complex task involves segmenting various structures from anatomical MRIs, numerical solution of the electromagnetic forward problem, signal denoising, a solution to the ill-posed electromagnetic inverse problem, and appropriate statistical control. This complexity not only constitutes methodological challenges to MEG investigators, but also offers a great deal of flexibility in data analysis. To successfully process M/EEG data, a comprehensive and well-documented analysis pipeline is therefore required.

MNE-Python is a sub-project of the more general academic software package MNE (Gramfort et al., 2013a), whose goal is to implement and provide a set of algorithms allowing users to assemble complete data analysis pipelines that encompass most phases of M/EEG data processing. Several of such software packages for M/EEG data processing exist, including Brainstorm (Tadel et al., 2011), EEGLAB [Delorme and Makeig (2004) and Delorme et al. (2011)], FieldTrip (Oostenveld et al., 2011), NutMeg (Dalal et al., 2011) and SPM (Litvak et al., 2011). These other packages are implemented in MATLAB, with some dependencies on external packages such as OpenMEEG (Gramfort et al., 2010b) for boundary element method (BEM) forward modeling or NeuroFEM for volume based finite element method (FEM) (Wolters et al., 2007) forward modeling. Many analysis methods are common to all these packages, yet MNE-Python offers some unique capabilities, in a coherent package facilitating the combination of standard and advanced techniques in a single environment described below.

While MNE-Python is designed to integrate with packages within the Python community, it also seamlessly interfaces with the other components of the MNE suite (and other M/EEG analysis tools) because it uses the same Neuromag FIF file format, with consistent analysis steps and compatible intermediate files. MNE-Python and the related MNE-Matlab sub-package that ship with MNE are both open source and distributed under the new BSD license, a.k.a 3-clause BSD, allowing their use in free as well as commercial software. The MNE-Python code is the most recent addition to the MNE suite. After an intensive collaborative software development effort, MNE-Python now provides a large number of additional features, such as time–frequency analysis, non-parametric statistics, connectivity estimation, independent component analysis (ICA), and decoding, a.k.a. multivariate pattern analysis (MVPA) or simply *supervised learning*, each of which is readily integrated into the standard MNE analysis pipeline. This comprehensive and still growing set of features available in the MNE-Python package is made possible by a group of dedicated contributors coming from multiple institutions, countries, and research areas of expertise who collaborate closely. These interactions are facilitated by the use of an inclusive, highly interactive software development process that is open for public viewing and contribution.

MNE-Python reimplements common M/EEG processing algorithms in pure Python. In addition, it also implements new algorithms, proposed and only recently published by the MNE-Python authors, making them publicly available for the first time (Gramfort et al., 2010a, 2011, 2013b; Larson and Lee, 2013). To achieve this task, MNE-Python is built on the foundation of core libraries provided by the scientific Python environment: *NumPy* (Van der Walt et al., 2011) offers the n-dimensional array data structure used to efficiently store and manipulate numerical data; *SciPy* is used mainly for linear algebra, signal processing and sparse matrices manipulation; *matplotlib* (Hunter, 2007) is used for 2D graphics; *Mayavi* (Ramachandran and Varoquaux, 2010) is employed for 3D rendering; *Scikit-Learn* [Pedregosa et al. (2011) and Buitinck et al. (2013)] is required for decoding tasks; and the *Python Data Analysis Library (Pandas)* is used for interfacing with spreadsheet table oriented data processing tools as often used in econometrics and behavioral sciences. *Mayavi, Scikit-Learn* and *Pandas* are only required by a small subset of the code, and are therefore considered optional dependencies. Besides these general libraries, MNE-Python has some other optional dependencies on neuroimaging packages such as Nibabel for reading and writing volume data (MRI, fMRI). The online documentation of MNE is generated with *Sphinx*http://sphinx-doc.org.

At present, MNE-Python contains more than 44,000 lines of Python code with around 22,000 lines of comments, contributed by a total of 35 persons.

In this paper, we describe the MNE-Python package in detail, starting from the standard analysis pipeline to more advanced usage. With this work, we aim to help standardize M/EEG analysis pipelines, to foster collaborative software development between institutes around the world, and consequently improve the reproducibility of M/EEG research findings.

## 2. The MNE-Python standard workflow for M/EEG data analysis

This section describes the standard analysis pipeline of MNE-Python. First, we discuss sample datasets that are available for working with MNE-Python. They allow readers to follow along with the workflow and examples in this manuscript. We then present the core Python structures employed in such an analysis, and use these to go from raw data preprocessing to the most commonly used linear inverse methods. The full script corresponding to the steps described below is available at the end of this section in Table 1.

**Table 1:** **From raw data to dSPM source estimates in less than 30 lines of code**.

import mne # *load data* raw = mne.fiff.Raw('raw.fif' ,preload=True) raw.info ['bads'] = ['MEG 2443', 'EEG 053'] #*mark bad channels* # *low-pass filter data* raw.filter (l_freq=None, h_freq=40.0) # *extract epochs and save them* picks = mne.fiff.pick_types (raw.info, meg=True, eeg=True, eog=True, exclude='bads') events = mne.find_events (raw) epochs = mne.Epochs (raw, events, event_id=1, tmin=−0.2, tmax=0.5, proj=True, picks=picks, baseline=(None, 0), preload=True, reject=dict (grad=4000e−13, mag=4e−12, eog=150e−6)) # *compute evoked response and noise covariance,and plot evoked* evoked = epochs.average () cov = mne.compute_covariance (epochs, tmax=0) evoked.plot () # *compute inverse operator* fwd_fname = 'sample_audvis−meg−eeg−oct−6−fwd.fif' fwd = mne.read_forward_solution(fwd_fname,surf_ori=True) inv = mne.minimum_norm.make_inverse_operator(raw.info, fwd, cov, loose=0.2) # *compute inverse solution* stc = mne.minimum_norm.apply_inverse(evoked, inv, lambda2=1./9., method='dSPM') # *morph it to average brain for group study and plot it* stc_avg = mne.morph_data ('sample', 'fsaverage', stc, 5, smooth=5) stc_avg.plot ()

### 2.1. Sample datasets

The MNE software package provides a sample dataset consisting of recordings from one subject with combined MEG and EEG conducted at the Martinos Center of Massachusetts General Hospital. These data were acquired with a Neuromag VectorView MEG system (Elekta Oy, Helsinki, Finland) with 306 sensors arranged in 102 triplets, each comprising two orthogonal planar gradiometers and one magnetometer. EEG was recorded simultaneously with 60 electrodes. In the experiment, auditory stimuli (delivered monaurally to the left or right ear) and visual stimuli (shown in the left or right visual hemifield) were presented in a random sequence with a stimulus-onset asynchrony (SOA) of 750 ms. To control for subject's attention, a smiley face was presented intermittently and the subject was asked to press a button upon its appearance. These data are used in the MNE-Python package and in this manuscript for illustration purposes. Small samples from these data are also used in the MNE-Python test suite which guarantees reproducibility of results across systems and environments, as well as the absence of regression when new code is contributed. This sample dataset can also serve as a standard validation dataset for M/EEG methods, hence favoring reproducibility of results. For the same purpose, MNE-Python facilitates easy access to the MEGSIM datasets (Aine et al., 2012) that include both experimental and simulated MEG data.

### 2.2. Design, application programming interface (API) and data structures

M/EEG data analysis typically involves three types of data containers coded in MNE-Python as Raw, Epochs, and Evoked objects. The raw data comes straight out of the acquisition system; these can be segmented into pieces often called epochs or trials, which generally correspond to segments of data after each repetition of a stimulus; these segments can be averaged to form evoked data. MNE-Python is designed to reproduce this standard operating procedure by offering convenient objects that facilitate data transformation.

Continuous raw data are stored in instances of the Raw class. MNE-Python supports reading raw data from various file formats e.g., BTI/4D, KIT, EDF, Biosemi BDF and BrainVision EEG. Other formats such as eXimia or CTF can be converted to FIF files using tools available in the MNE-C package, also available at http://martinos.org/mne. The Neo project (Garcia et al., under review) implements readers in Python for micromed and elan files, which can facilitate the use of these formats with MNE-Python. The FIF file format allows organization of any type of information into a multi-leaved tree structure of elements known as *tags*. It is at the core of the MNE-Python package which favored the development of highly optimized reading and writing routines for this format. It offers for example the ability to read data from disk only when needed. This access-on-demand principle can also be inherited by other classes that build upon Raw (such as Epochs and Evoked, below), which offers the possibility to process data with a very limited memory usage. Typical processing steps at this stage include filtering, noise suppression (such as blinks or cardiac artifacts), data cropping, and visual data exploration. All of these are supported by convenient instance methods of the Raw class that will be explored in greater detail below.

Typical M/EEG experiments involve presentation of stimuli and responses based on some form of task demands. The occurrence of each stimulus or response can can be used to define an epoch which captures the brain signals preceding the stimulus or response as well as the response following them. Depending on the experimental paradigm and the analysis employed, an epoch is typically 500 ms to 2 s long. Epochs of different experimental conditions obtained from one subject are stored in MNE-Python in an instance of the Epochs class. An Epochs instance is created by specifying one or more instances of Raw to operate on, the event/stimulus type(s) of interest, and the time window to include. The Epochs object has various parameters for preprocessing single trial data, such as baseline correction, signal detrending, and temporal decimation. Epochs can be averaged to form evoked data containing the MEG and EEG signals known respectively as event related fields (ERFs) and event related potentials (ERPs). The averaged data are stored in instances of the Evoked class, and can be created simply by calling the average method on an instance of Epochs. As the Raw class, both Epochs and Evoked classes expose convenient plot methods that help visualizing single trials and evoked responses.

Each of these data containers can be written to and read from disk using the FIF file format, which is readable from the MNE C code and the MNE-Matlab toolbox. These containers share some common attributes such as ch_names, which is a Python list containing the names of all of the channels, and an info attribute which is a modified Python dictionary storing all the metadata about the recordings. This attribute is commonly called the *measurement information*. For example, the sfreq key in the info dictionary, accessed with info['sfreq'] syntax, is the sampling frequency; the channel types and positions are available in info['chs']; the positions of the head digitization points used for coregistration are contained in info['dig']; and info['bads'] stores the list of bad channels. The info attribute can be used to conveniently do some channel selection by type (e.g., gradiometers, magnetometers, EEG), general position (e.g., right temporal channels), or simply by channel names. These convenience functions in MNE-Python are known as *pick* functions, and they start with pick_ (e.g., pick_types to select by channel type). Other standard data structures in MNE-Python handle forward operators, covariance matrices, independent components, and source estimates. These structures will be introduced below after explaining their role in the standard pipeline. Importantly, the API follows as much as possible the Python standard library and the widely spread NumPy package. It avoids the proliferation of classes and limits the use of complex inheritance mechanisms. This helps to keep the code simple, favoring new contributions.

### 2.3. Preprocessing

The major goal when preprocessing data is to attenuate noise and artifacts from exogenous (environmental) and endogenous (biological) sources. Noise reduction strategies generally fall into two broad categories: exclusion of contaminated data segments and attenuation of artifacts by use of signal-processing techniques (Gross et al., 2013). MNE-Python offers both options at different stages of the pipeline, through functions for automatic or semi-automatic data preprocessing as well as interactive plotting capabilities.

The first preprocessing step often consists in restricting the signal to a frequency range of interest through filtering. MNE-Python supports band-pass, low-pass, high-pass, band-stop, and notch filtering. Instances of Raw can be filtered using the filter method that supports fast Fourier transform (FFT) based finite impulse response (FIR) filters (optionally using the overlap-add technique to minimize computation time), as well as infinite impulse response (IIR) filters such as Butterworth filters implemented in SciPy. Several channels can be filtered in parallel, thanks to the standard *multiprocessing* Python module exposed via the Joblib package (http://pythonhosted.org/joblib/). The FFTs used to implement FIR filters can also be efficiently computed on the graphical processing unit (GPU) via CUDA and PyCUDA (Klöckner et al., 2012), further reducing the execution time.

When segmenting continuous data into epochs, single epochs can be rejected based on visual inspection, or automatically by defining thresholds for peak-to-peak amplitude and flat signal detection. The channels contributing to rejected epochs can also be visualized to determine whether bad channels have been missed by visual inspection, or if noise rejection methods have been inadequate.

**Figure 1:**
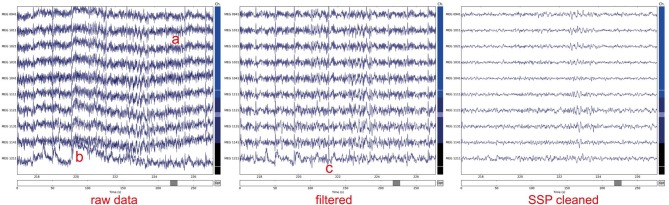
**Noisy raw MEG magnetometer signals corrupted by a) slow drifts, b) line noise (at 50 or 60 Hz), and c) heartbeats present across sensors**. To clean signals data were filtered between 1 and 45 Hz. Subsequently, five signal space projection (SSP) vectors were applied (3 computed from empty room noise, 2 from ECG signals). The plots were generated using the plot method of the Raw class.

**Figure 2:**
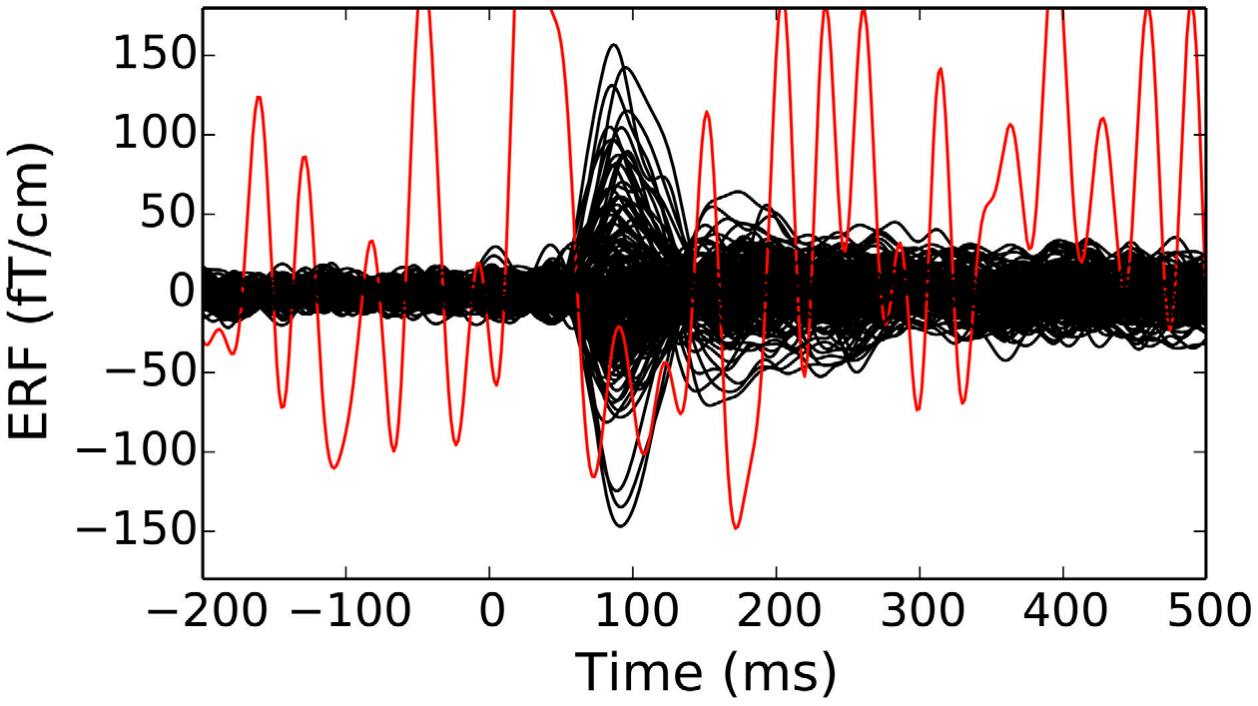
**An evoked response (event-related fields in planar gradiometers of an Elekta-Neuromag Vectorview system) showing traces for individual channels (bad channels are colored in red)**. Epochs with large peak-to-peak signals as well as channels marked as bad can be discarded from further analyses. The figure was generated using the plot method of the Evoked class.

Instead of simply excluding contaminated data from the analysis, artifacts can sometimes be removed or significantly suppressed by using methods for signal decomposition such as signal space projection (SSP; Uusitalo and Ilmoniemi, 1997) or independent component analysis (ICA, see Section 3.1 below). The assumption behind the SSP method is that artifacts are confined to a small-dimensional spatial subspace with specific topographic patterns that are orthogonal or almost orthogonal to the brain signal patterns of interest and can thus be suppressed with appropriate projection vectors. Projection vectors can be derived from instances of Raw as well as Epochs. MNE-Python also offers command-line level scripts and Python-level functions to automatically detect heart beats and eye blinks in the data, making automatic SSP computation possible. Once projection vectors are specified for subtraction in the measurement info, MNE minimizes memory and disk space usage by not modifying the original data but instead applying the projections on demand. This enables the user to explore the effects of particular SSPs later in the pipeline and to selectively abandon some projection vectors if the signals of interest are attenuated. After the above steps, one can obtain clean data as illustrated in Figure 1, which then can be further processed in epochs and evoked data, see Figure 2.

### 2.4. Linear inverse methods

After performing noise reduction via preprocessing, sensor-level data, especially those from planar gradiometers, may indicate the probable number and approximate locations of active sources. In order to actually locate the sources, several different unique solutions to the ill-posed electromagnetic inverse problem exist. Each localization technique has its own modeling assumptions and thus also strengths and limitations. Therefore, the MNE software provides a selection of inverse modeling approaches. Importantly, in all of the approaches discussed here, the elementary source employed is a current dipole, motivated by the physiological nature of the cerebral currents measurable in M/EEG (Hämäläinen et al., 1993). Different source modeling approaches are set apart by the selection constraints on the sources and other criteria to arrive at the *best estimate* for the cerebral current distribution as a function of time.

Source localization methods generally fall into one of three categories: parametric overdetermined methods such as *time-varying dipole fitting* (Scherg and Von Cramon, 1985), scanning methods (including beamformers and the MUSIC algorithm), and distributed inverse methods. While MNE-Python does not provide dipole-fitting functionality, it does implement multiple beamformer methods and distributed inverse methods. The most popular of these is the software namesake MNE, which stands for Minimum-Norm Estimate (Wang et al., 1992; Hämäläinen and Ilmoniemi, 1994), and its variants which include dSPM (Dale et al., 2000) and sLORETA (Pascual-Marqui, 2002).

The standard MNE pipeline by uses MNE or dSPM as the inverse method by default. These methods employ the (weighted) ℓ_2_-norm of the current distribution as regularizer. The important practical benefit of such ℓ_2_ solvers is that the inverse problem is linear and, therefore, the solution is obtained by multiplying the data with a matrix, called the *inverse operator*. Once the inverse operator has been constructed, it can be applied to evoked, epochs, and raw data containers. The output of these inverse solvers, as well as all alternative inverse methods, is provided as instances of the SourceEstimate object that can be saved to disk as .stc files. The acronym *stc* stands for source times courses.

**Figure 3:**
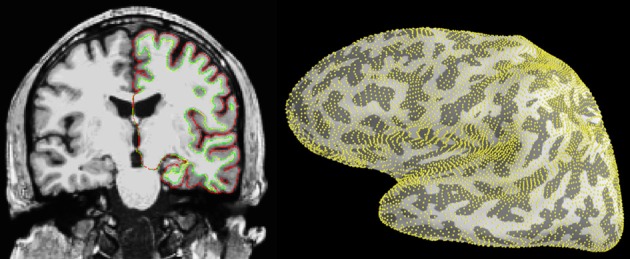
**Cortical segmentation used for the source space in the distributed model with MNE**. **Left:** The pial (red) and white matter (green) surfaces overlaid on an MRI slice. **Right:** The right-hemisphere part of the source space (yellow dots), represented on the inflated surface of the left hemisphere, was obtained by subdivision of an icosahedron leading to 10242 locations per hemisphere with an average nearest-neighbor distance of 3.1 mm. Left image was produced with FreeSurfer tksurfer tool and the right one with *PySurfer* (http://pysurfer.github.io) which internally depends on *Mayavi* (Ramachandran and Varoquaux, 2010).

**Figure 4:**
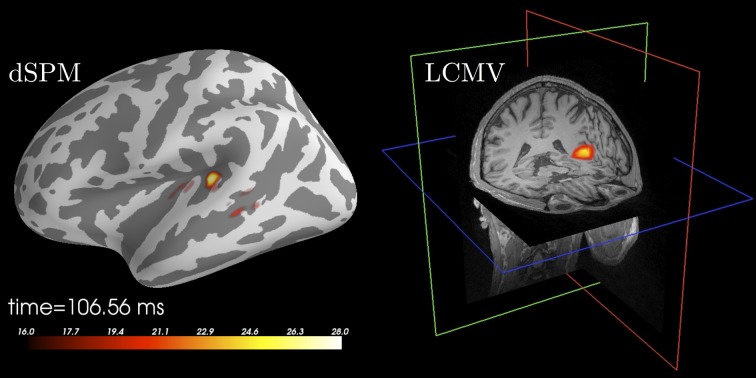
**Source localization of an auditory N100 component**. **Left:** Results obtained using dSPM and a surface source space based on combined MEG and EEG data. The figure was generated using the plot method of the SourceEstimate class which internally calls *PySurfer*. **Right:** Results obtained using LCMV beamformer and a volume source space based on MEG channels only. The figure was generated using Freeview shipped with FreeSurfer.

The source estimates are defined on what is called a *source space*, which specifies the locations of the candidate dipole sources, typically regularly sampled over the cortical mantle or on a volumetric grid. The source space routinely used by MNE is based on the surface defined by the boundary between the gray and the white matter, which consists of a high-resolution mesh with over 100,000 vertices per hemisphere. To reduce the number of dipoles in the source space defined on this surface, it is necessary to decimate the mesh. However, preserving surface topology, spacing, and neighborhood information between neighboring vertices is difficult. Therefore, MNE uses a subsampling strategy that consists of polygon subdivisions using the spherical coordinate system provided by FreeSurfer. For example, an icosahedron subdivided 5 times, abbreviated ico-5, consists of 10242 locations per hemisphere, which leads to an average spacing of 3.1 mm between dipoles (assuming a reasonable surface area of 1000 cm^2^ per hemisphere), see illustration in Figure 3. The source estimate defined on this low-resolution surface can then be up-sampled and represented on the original high-resolution cortical surface as presented in Figure 4.

### 2.5. Surface-based normalization

**Figure 5:**
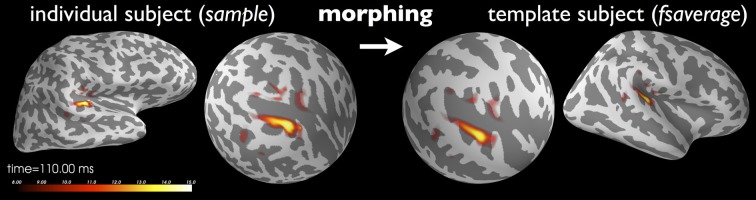
**Current estimates obtained from an individual subject can be remapped (morphed), i.e., normalized, to another cortical surface, such as that of the FreeSurfer average brain “*fsaverage*” shown here**. The normalization is done separably for both hemispheres using a non-linear registration procedure defined on the sphere (Dale et al., 1999; Fischl et al., 1999). Here, the N100m auditory evoked response is localized using dSPM and then mapped to “fsaverage.” Images were produced with *PySurfer*.

While clinical examinations generally consider data from each patient separately, neuroscience questions are frequently answered by comparing and combining data from groups of subjects. To achieve this, data from all participating subjects need to be transformed to a common space in a manner that helps compensate for inter-subject differences. This procedure, called *morphing* by the MNE software, exploits the FreeSurfer spherical coordinate system defined for each hemisphere (Dale et al., 1999; Fischl et al., 1999). The process is illustrated in Figure 5.

## 3. Advanced examples

Having described the standard MNE-Python workflow for source localization, we will now present some more advanced examples of data processing. Some of these examples provide alternative options for preprocessing and source localization.

### 3.1. Denoising with independent component analysis (ICA)

**Figure 6:**
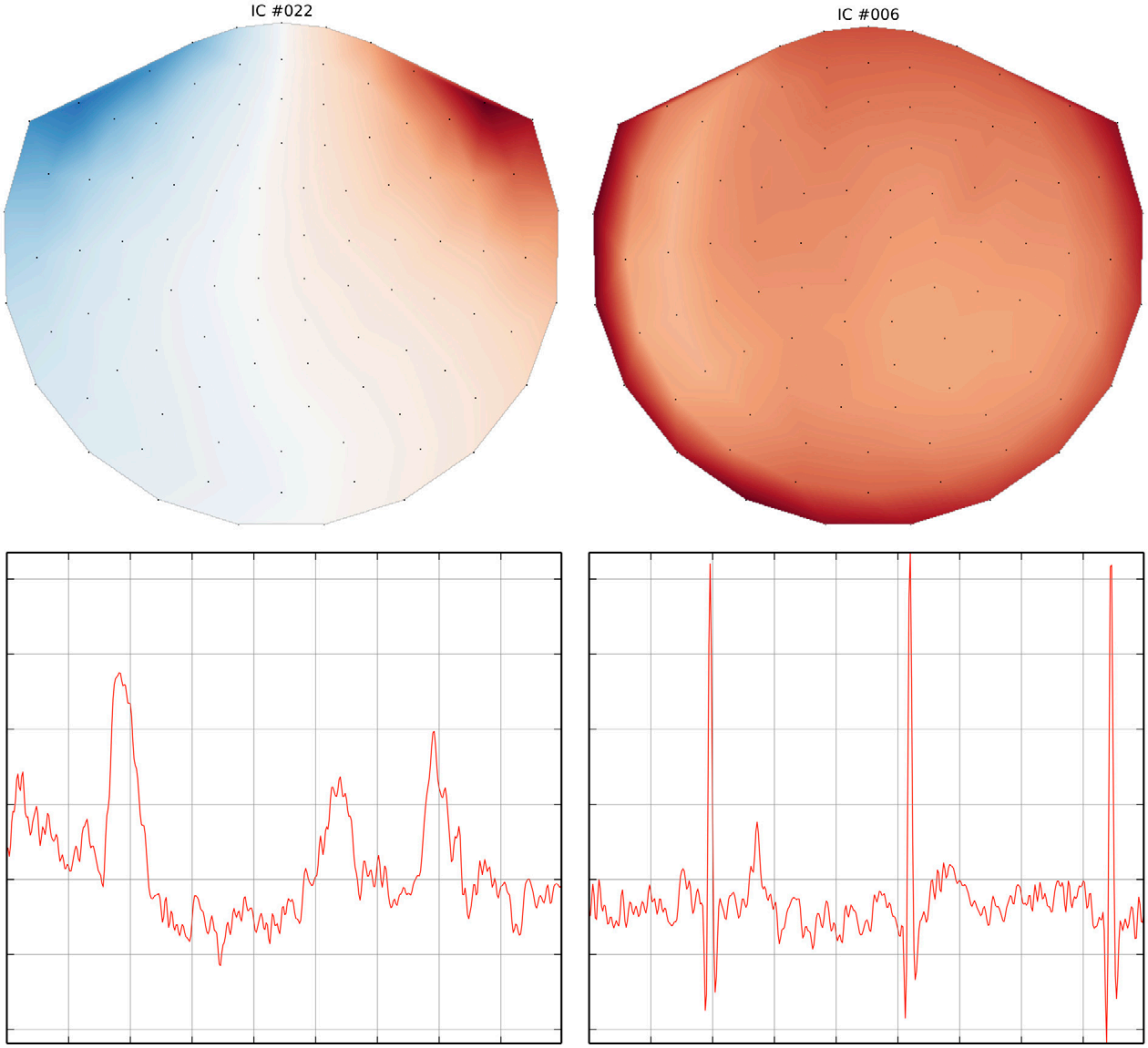
**Topographic and trellis plots of two automatically identified ICA components**. The component #22 corresponds to the EOG artifact with a topography on the magnetometers showing frontal signals and a waveform typical of an eye blink. The component #6 on the right captures the ECG artifact with a waveform matching 3 heart beats.

In addition to SSP, MNE supports identifying artifacts and latent components using temporal ICA. This method constitutes a latent variable model that estimates statistically independent sources, based on distribution criteria such as kurtosis or skewness. When applied to M/EEG data, artifacts can be removed by zeroing out the related independent components before inverse transforming the latent sources back into the measurement space. The ICA algorithm currently supported by MNE-Python is FastICA (Hyvärinen and Oja, 2000) implemented in *Scikit-Learn* (Pedregosa et al., 2011). Here, MNE-Python has added a domain specific set of convenience functions covering visualization, automated component selection, persistence as well as integration with the MNE-Python object system. ICA in MNE-Python is handled by the ICA class which allows one to fit an unmixing matrix on either Raw or Epochs by calling the related decompose_raw or decompose_epochs methods. After a model has been fitted, the resulting source time series can be visualized using trellis plots (Becker et al., 1996) (cf. Figure 6) as provided by the plot_sources_raw and plot_sources_epochs methods (illustrated in Figure 6). In addition, topographic plots depicting the spatial sensitivities of the unmixing matrix are provided by the plot_topomap method (illustrated in Figure 6). Importantly, the find_sources_raw and find_sources_epochs methods allow for identifying sources based on bivariate measures, such as Pearson correlations with ECG recording, or simply based on univariate measures such as variance or kurtosis. The API, moreover, supports user-defined scoring measures. Identified source components can then be marked in the ICA object's exclude attribute and saved into a FIF file, together with the unmixing matrix and runtime information. This supports a sustainable, demand-driven workflow: neither sources nor cleaned data need to be saved, signals can be reconstructed from the saved ICA structure as required. For advanced use cases, sources can be exported as regular raw data or epochs objects, and saved into FIF files (sources_as_raw and sources_as_epochs). This allows any MNE-Python analysis to be performed on the ICA time series. A simplified ICA workflow for identifying, visualizing and removing cardiac artifacts is illustrated in Table 2.

**Table 2:** **From epochs to ICA artifact removal in less than 20 lines of code**.

import mne from mne.datasets import sample import numpy as np # *Setup paths and prepare data* raw_fname = sample.data_path () + '/MEG/sample/sample_audvis_filt-0-40_raw.fif' raw = mne.fiff.Raw (raw_fname) picks = mne.fiff.pick_types (raw.info, meg='mag', exclude='bads') ica = mne.preprocessing.ICA (n_components=49) ica.decompose_raw (raw, picks=picks, decim=3) # *use every third sample* # *find artifacts using bivariate and univariate measures* scores = ica.find_sources_raw (raw, target='EOG 061', score_func='correlation') ica.exclude += [scores.argmax ()] scores = ica.find_sources_raw (raw, score_func=np.var) ica.exclude += [scores.argmax ()] # *Visualize result using topography and source time course* ica.plot_topomap (ica.exclude) ica.plot_sources_raw (raw, ica.exclude, start=100., stop=103.)

### 3.2. Non-parametric cluster-level statistics

For traditional cross-subject inferences, MNE-Python offers several parametric and non-parametric statistical methods. Parametric statistics provide valid statistical contrasts in so far as the data under test conform to certain underlying assumptions of Gaussianity. The more general class of non-parametric statistics, which we will focus on here, do not require such assumptions to be satisfied (Nichols and Holmes, 2002; Pantazis et al., 2005).

M/EEG data naturally contains spatial correlations, whether the signals are represented in sensor space or source space, as temporal patterns or time–frequency representations. Moreover, due to filtering and even the characteristics of the signals themselves, there are typically strong temporal correlations as well. Mass univariate methods provide statistical contrasts at each “location” across all dimensions, e.g., at each spatio-temporal point in a cortical temporal pattern, independently. However, due to the highly correlated nature of the data, the resulting Bonferroni or false discovery rate corrections (Benjamini and Hochberg, 1995) are generally overly conservative. Moreover, making inferences over individual spatio-temporal (or other dimensional) points is typically not of principal interest. Instead, studies typically seek to identify contiguous regions within some particular dimensionality, be it spatio-temporal or time–frequency, during which activation is greater in one condition compared to a baseline or another condition. This leads to the use of cluster-based statistics, which seek such contiguous regions of significant activation (Maris and Oostenveld, 2007).

**Figure 7:**
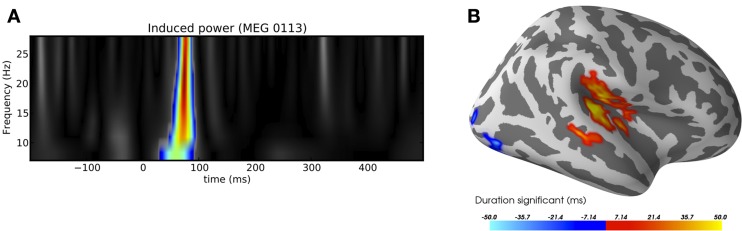
**Examples of clustering**. **(A)** Time-frequency clustering showing a significant region of activation following an auditory stimulus. **(B)** A visualization of the significant spatio-temporal activations in a contrast between auditory stimulation and visual stimulation using the sample dataset. The red regions were more active after auditory than after visual stimulation, and vice-versa for blue regions. Image **(B)** was produced with *PySurfer*.

MNE-Python includes a general framework for cluster-based tests to allow for performing arbitrary sets of contrasts along arbitrary dimensions while controlling for multiple comparisons. In practice, this means that the code is designed to work with many forms of data, whether they are stored as SourceEstimate for source-space data, or as Evoked for sensor-space data, or even as custom data formats, as necessary for time–frequency data. It can operate on any *NumPy* array using the natural (grid) connectivity structure, or a more complex connectivity structure (such as those in a brain source space) with help of a sparse adjacency matrix. MNE-Python also facilitates the use of methods for variance control, such as the “hat” method (Ridgway et al., 2012). Two common use cases are provided in Figure 7.

### 3.3. Decoding—MVPA—supervised learning

**Figure 8:**
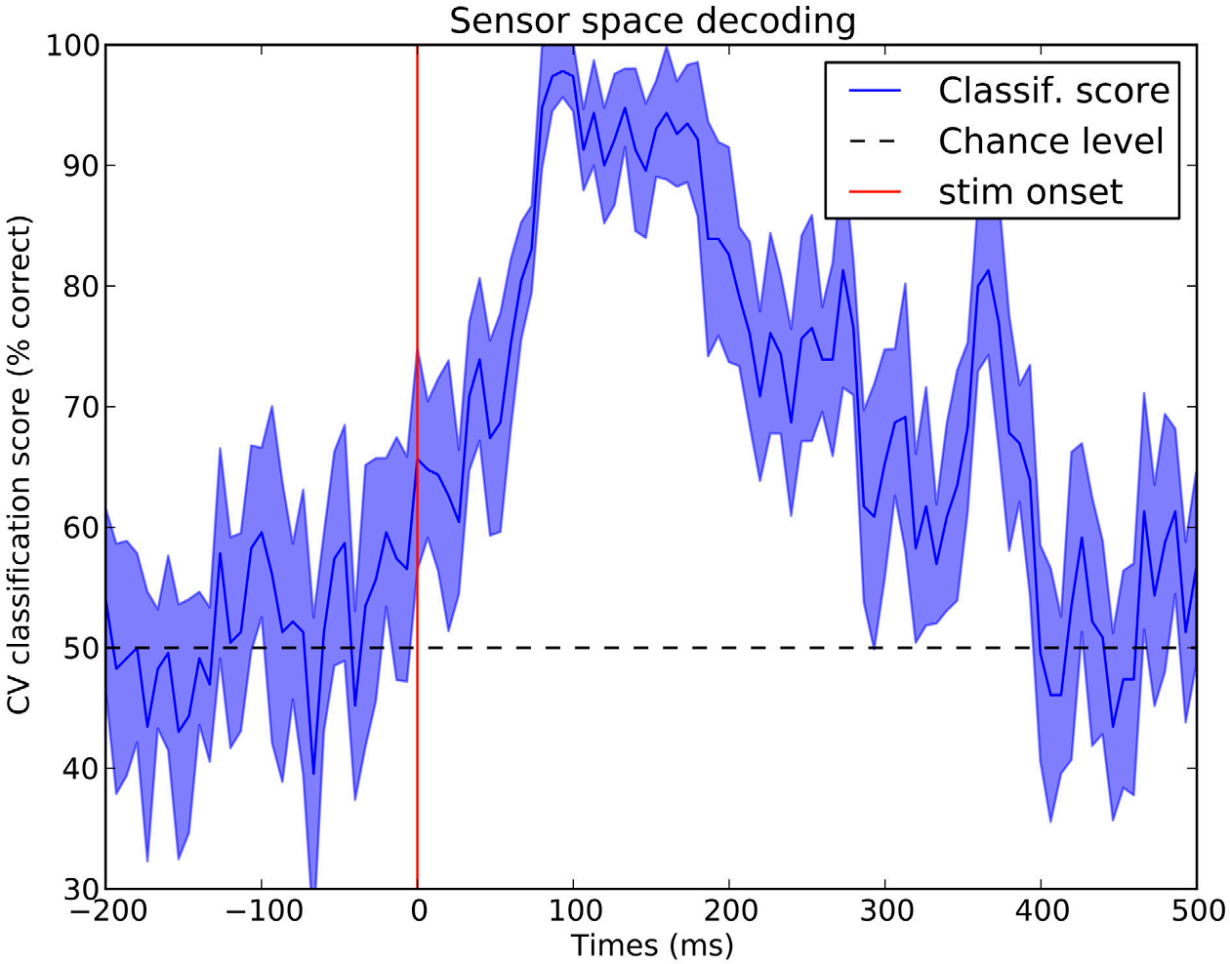
**Sensor space decoding**. At every time instant, a linear support vector machine (SVM) classifier is used with a cross-validation loop to test if one can distinguish data following a stimulus in the left ear or in the left visual field. One can observe that the two conditions start to be significantly differentiated as early as 50 ms and maximally at 100 ms which corresponds to the peak of the primary auditory response. Such a statistical procedure is a quick and easy way to see in which time window the effect of interest occurs.

MNE-Python can easily be used for decoding using *Scikit-Learn* (Pedregosa et al., 2011). Decoding is often referred to as multivariate pattern analysis (MVPA), or simply supervised learning. Figure 8 presents cross-validation scores in a binary classification task that consists of predicting, at each time point, if an epoch corresponds to a visual flash in the left hemifield or a left auditory stimulus. Results are presented in Figure 8. The script to reproduce this figure is available in Table 3.

**Table 3:** **Sensor space decoding of MEG data**.

import mne from sklearn.svm import SVC from sklearn.cross_validation import cross_val_score, ShuffleSplit # *Take only the data channels (here the gradiometers)* data_picks = mne.fiff.pick_types (epochs1.info, meg='grad', exclude='bads') # *Make arrays X and y such that:* # *X is 3d with X.shape[0] is the total number of epochs to classify* # *y is filled with integers coding for the class to predict* # *We must have X.shape[0] equal to y.shape[0]* X = [e.get_data () [:, data_picks, :] for e in (epochs1, epochs2)] y = [k * np.ones (len(this_X)) for k, this_X in enumerate(X)] X = np.concatenate(X) y = np.concatenate(y) clf = SVC(C=1, kernel='linear') # *Define a monte-carlo cross-validation generator (to reduce variance):* cv = ShuffleSplit (len(X), 10, test_size=0.2) scores, std_scores = np.empty (X.shape[2]), np.empty (X.shape[2]) for t in xrange(X.shape[2]): Xt = X[:, :, t] scores_t = cross_val_score(clf, Xt, y, cv=cv, n_jobs=1) scores [t] = scores_t.mean () std_scores [t] = scores_t.std ()

### 3.4. Functional connectivity

Functional connectivity estimation aims to estimate the structure and properties of the network describing the dependencies between a number of locations in either sensor- or source-space. To estimate connectivity from M/EEG data, MNE-Python employs single-trial responses, which enables the detection of relationships between time series that are consistent across trials. Source-space connectivity estimation requires the use of an inverse method to obtain a source estimate for each trial. While computationally demanding, estimating connectivity in source-space has the advantage that the connectivity can be more readily related to the underlying anatomy, which is difficult in the sensor space.

The connectivity module in MNE-Python supports a number of bivariate spectral connectivity measures, i.e., connectivity is estimated by analyzing pairs of time series, and the connectivity scores depend on the phase consistency across trials between the time series at a given frequency. Examples of such measures are coherence, imaginary coherence (Nolte et al., 2004), and phase-locking value (PLV) (Lachaux et al., 1999). The motivation for using imaginary coherence and related methods is that they discard or downweight the contributions of the real part of the cross spectrum and, therefore, zero-lag correlations, which can be largely a result of the spatial spread of the measured signal or source estimate distributions (Schoffelen and Gross, 2009). However, note that even though some methods can suppress the effects of the spatial spread, connectivity estimates should be interpreted with caution; due to the bivariate nature of the supported measures, there can be a large number of apparent connections due to a latent region connecting or driving two regions that both contribute to the measured data. Multivariate connectivity measures, such as partial coherence (Granger and Hatanaka, 1964), can alleviate this problem by analyzing the connectivity between all regions simultaneously (cf. Schelter et al., 2006). We plan to add support for such measures in the future.

The connectivity estimation routines in MNE-Python are designed to be flexible yet computationally efficient. When estimating connectivity in sensor-space, an instance of Epochs is used as input to the connectivity estimation routine. For source-space connectivity estimation, a Python list containing SourceEstimate instances is used. Instead of a list, it is also possible to use a Python generator object which produces SourceEstimate instances. This option drastically reduces the memory requirements, as the data is read on-demand from the raw file and projected to source-space during the connectivity computation, therefore requiring only a single SourceEstimate instance to be kept in memory. To use this feature, inverse methods which operate on Epochs, e.g., apply_inverse_epochs, have the option to return a generator object instead of a list. For linear inverse methods, e.g., MNE, dSPM, sLORETA, further computational savings are achieved by storing the inverse kernel and sensor-space data in the SourceEstimate objects, which allows the connectivity estimation routine to exploit the linearity of the operations and apply the time-frequency transforms before projecting the data to source-space.

**Figure 9:**
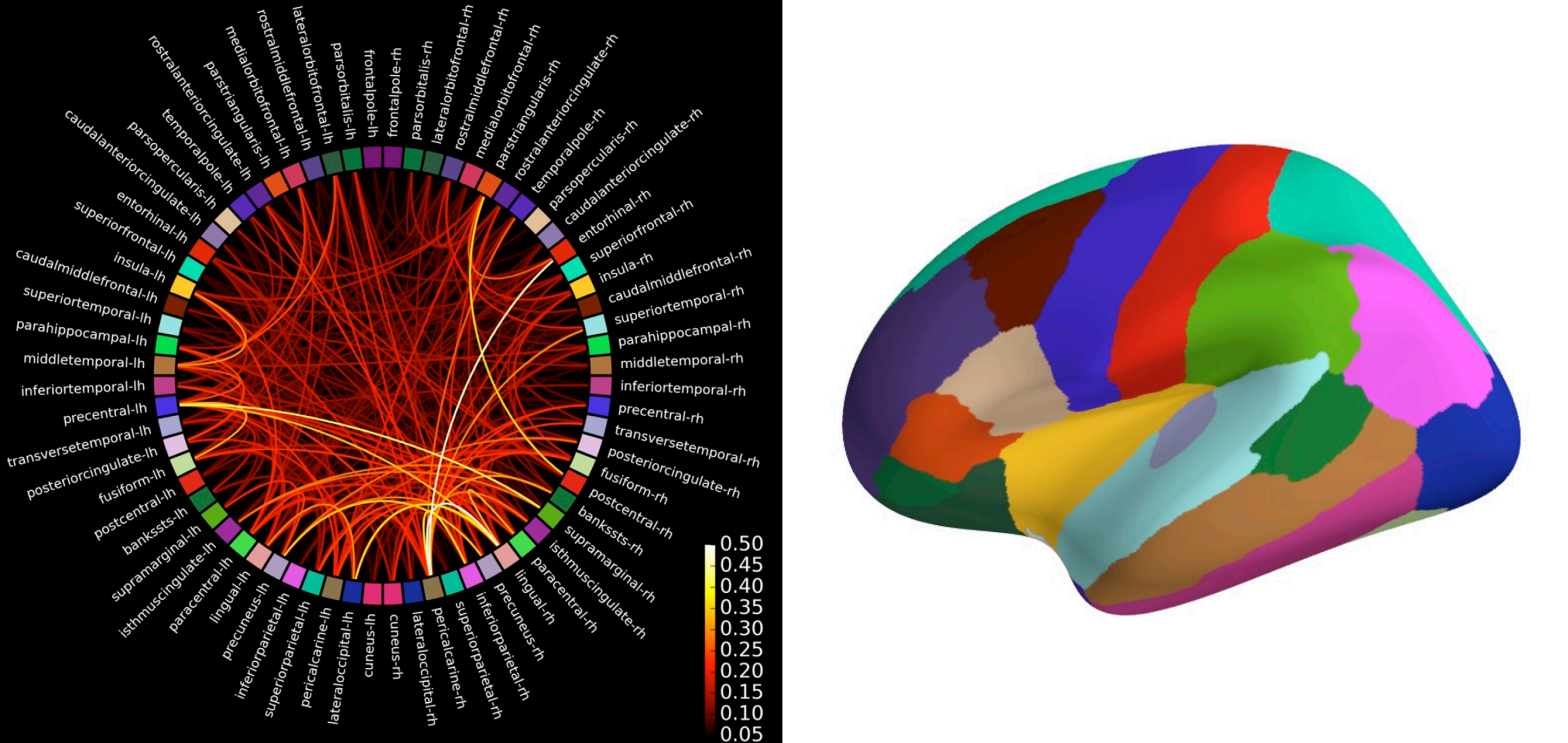
**Connectivity between brain regions of interests, also called labels, extracted from the automatic FreeSurfer parcellation visualized using plot_connectivity_circle**. The image of the right presents these labels on the inflated cortical surface. The colors are in agreement between both figures. Left image was produced with *matplotlib* and right image with *PySurfer*.

Due to the large number of time series, connectivity estimation between all pairs of time series in source-space is computationally demanding. To alleviate this problem, the user has the option to specify pairs of signals for which connectivity should be estimated, which makes it possible, for example, to compute the connectivity between a seed location and the rest of the brain. For all-to-all connectivity estimation in source-space, an attractive option is also to reduce the number of time series, and thus the computational demand, by summarizing the source time series within a set of cortical regions. We provide functions to do this automatically for cortical parcellations obtained by FreeSurfer, which employs probabilistic atlases and cortical folding patterns for an automated subject-specific segmentation of the cortex into anatomical regions (Fischl et al., 2004; Desikan et al., 2006; Destrieux et al., 2010). The obtained set of summary time series can then be used as input to the connectivity estimation. The association of time series with cortical regions simplifies the interpretation of results and it makes them directly comparable across subjects since, due to the subject-specific parcellation, each time series corresponds to the same anatomical region in each subject. Code to compute the connectivity between the labels corresponding to the 68 cortical regions in the FreeSurfer “aparc” parcellation is shown in Table 4 and the results are shown in Figure 9.

**Table 4:** **Connectivity estimation between cortical regions in the source space**.

import mne from mne.minimum_norm import apply_inverse_epochs from mne.connectivity import spectral_connectivity # *Apply inverse to single epochs* stcs = apply_inverse_epochs (epochs, inverse_op, lambda2, method='dSPM', pick_normal=True, return_generator=True) # *Summarize souce estimates in labels* labels, label_colors = mne.labels_from_parc ('sample', parc='aparc', subjects_dir=subjects_dir) label ts = mne.extract_label_time_course (stcs, labels, inverse_op ['src'], mode='mean_flip', return_generator=True) # *Compute all-to-all connectivity between labels* con, freqs, times, n_epochs, n_tapers = spectral_connectivity (label_ts, method='wpli2_debiased', mode='multitaper', sfreq=raw.info['sfreq'], fmin=8., fmax=13., faverage=True, mt_adaptive=True)

### 3.5. Beamformers

MNE-Python implements two source localization techniques based on beamforming: Linearly-Constrained Minimum Variance (LCMV) in the time domain (Van Veen et al., 1997) and Dynamic Imaging of Coherent Sources (DICS) in the frequency domain (Gross et al., 2001). Beamformers construct adaptive spatial filters for each location in the source space given a data covariance (or cross-spectral density in DICS). This leads to pseudo-images of “source power” that one can store as SourceEstimates.

Figure 4 presents example results of applying the LCMV beamformer to the sample data set for comparison with results achieved using dSPM. The code that was used to generate this example is listed in Table 5.

**Table 5:** **Inverse modeling using the LCMV beamformer**.

import mne # *load raw data and create epochs and evoked objects as in Table 1, but picking* # *only MEG channels using mne.fiff.pick_types(raw.info, meg=True, eeg=False)* # *compute noise and data covariance* noise_cov = mne.compute_covariance(epochs, tmax=0.0) noise_cov = mne.cov.regularize (noise_cov, evoked.info, mag=0.05, grad=0.05, eeg=0.1, proj=True) data_cov = mne.compute_covariance (epochs, tmin=0.04, tmax=0.15) # *compute LCMV inverse solution* fwd_fname = 'sample_audvis-meg-vol-7-fwd.fif' fwd = mne.read_forward_solution (fwd_fname, surf_ori=True) stc = mne.beamformer.lcmv(evoked, fwd, noise_cov, data_cov, reg=0.1, pick_ori='max-power') # *save result in 4D nifti file for plotting with Freesurfer* stc.save_as_volume('lcmv.nii.gz', fwd['src'], mri_resolution=False)

### 3.6. Non-linear inverse methods

**Figure 10:**
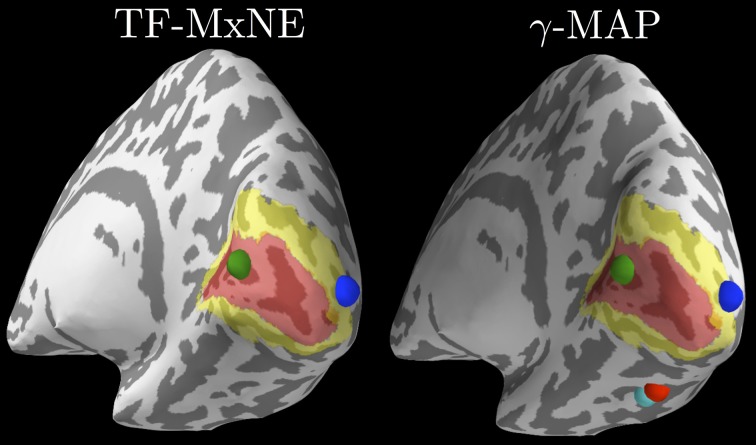
**Source localization with non-linear sparse solvers**. The left plot shows results from TF-MxNE on raw unfiltered data (due to the built-in temporal smoothing), and the right plot shows results from γ-MAP on the same data but filtered below 40 Hz. One can observe the agreement between both methods on the sources in the primary (red) and secondary (yellow) visual cortices delineated by FreeSurfer using an atlas. The γ-MAP identifies two additional sources in the right fusiform gyrus along the visual ventral stream. These sources that would not be naturally expected from such simple visual stimuli are weak and peak later in time, which makes them nevertheless plausible.

All the source estimation strategies presented thus far, from MNE to dSPM or beamformers, lead to linear transforms of sensor-space data to obtain source estimates. There are also multiple inverse approaches that yield non-linear source estimation procedures. Such methods have in common to promote spatially sparse estimates. In other words, source configurations consisting of a small set of dipoles are favored to explain the data. MNE-Python implements three of these approaches, namely mixed-norm estimates (MxNE) (Gramfort et al., 2012), time–frequency mixed-norm estimates (TF-MxNE) (Gramfort et al., 2013b) that regularize the estimates in a time–frequency representation of the source signals, and a sparse Bayesian learning technique named γ-MAP (Wipf and Nagarajan, 2009). Source localization results obtained on the ERF evoked by the left visual stimulus with both TF-MxNE and γ-MAP are presented in Figure 10.

## 4. Discussion

Data processing, such as M/EEG analysis, can be thought of as a sequence of operations, where each step has an impact on the subsequent (and ultimately final) results. In the preceding sections we have first detailed the steps of the standard MNE pipeline, followed by the presentation of some alternative and complementary analysis tools made available by the package.

MNE-Python is a scripting-based package with many visualization capabilities for visualizing results of processing steps and final outputs, but limited graphical user interfaces (GUIs) for actually performing processing steps. Leveraging the good readability on the Python language, particular care has been taken to keep the scripts simple, easy to read and to write. This is similar in spirit with the FieldTrip package (Oostenveld et al., 2011), in that it pushes users toward standardizing analyses via scripting instead of processing data in a GUI. Perhaps the largest downside of this scripting approach is that users clearly need to be able to write reasonable scripts. However this approach, which is facilitated by many examples that can be copied from the MNE website, has very clear benefits. First, our experience from analyzing several M/EEG studies unambiguously indicates that the processing pipeline must be tailored for each study based on the equipment used, the nature of the experiment, and the hypotheses under test. Even though most pipelines follow the same general logic (filtering, epoching, averaging, etc.), the number of options is large even for such standard steps. Scripting gives the flexibility to set those options once per study to handle the requirements of different M/EEG studies. Second, analyses conducted with help of documented scripts lead to more reproducible results and ultimately help improve the quality of the research results. Finally, studies that involve processing of data from dozens or hundreds of subjects are made tractable via scripting. This is particularly relevant in an era of large-scale data analysis with possibly more than a thousand subjects, *cf.*, the Human Brain Project or the Human Connectome Project (Van Essen et al., 2012).

Software-based data analysis is not limited to neuroimaging, and the fact today is that neuroscientists from different academic disciplines spend an increasing amount of time writing software to process their experimental data. We would wager that almost all scientific data are ultimately processed with computers software. The practical consequence of this is that the quality of the science produced relies on the quality of the software written (Dubois, 2005). The success of digital data analysis is made possible not just by acquiring high-quality data and sometimes by using sophisticated numerical and mathematical methods, but critically it is made possible by using correct implementations of methods. The MNE-Python project is developed and maintained to help provide the best quality in terms of accuracy, efficiency, and readability. In order to preserve analysis accuracy, the development process requires the writing of unit and regression tests (so-called test-driven development) that ensure that the software is installed and functioning correctly, yielding results that match those previously obtained from many different users and machines. This testing framework currently covers about 86% of the lines of MNE-Python code, which not only enhances the quality and stability of the software but also makes it easier to incorporate new contributions quickly without breaking existing code. Code quality is also improved by a *peer review* process among the developers. Any code contribution must be read by at least two people, the author and a reviewer, in order to mitigate the risk of errors. Moreover, the entire source code and full development history is made publicly available by a distributed version control system. This makes it possible to keep track of the development of the project and handle code changes in a way that minimizes the risk of rendering existing scripts and analysis pipelines inoperable. Finally, large parts of the source code are commented using inline documentation that allows for automatically building user manuals in PDF and HTML formats. The *Ohloh.net*^1^ source code analysis project attests that 35% of the source code consists of documentation and with this, MNE-Python scores in the upper third of the most well documented Python projects.

Some recent studies have pointed out the heterogeneity of functional MRI data analysis pipelines (Carp, 2012a). These studies quantify the combinatorial explosion of analysis options when many different steps are combined as required when analyzing neuroimaging data. Although they focused on fMRI, the same issue arises for M/EEG. We argue that this fact does not need to become a significant drawback, as long as the details required to make the analysis reproducible are available. A difficulty does arise in that whatever level of detail is provided in a methods section of a paper, it is ultimately unlikely to be sufficient to provide access to all parameters used. However, sharing the proper code provides a better guarantee for reproducible science. The previously mentioned studies also raise the issue that the geographical location of the investigators biases their choice in terms of method and software. Again, this is not wrong *per se*, as expertise is more easily found from colleagues than mailing lists or user documentation. By favoring on-line collaborative work between international institutions, MNE-Python aims to reduce this geographical bias.

While an important goal in science is the reproducibility of results, reproducibility can have two levels of meaning. Rerunning the same analysis (code) on the same dataset using the same machine should always be possible. However, we should really be aiming for a deeper level of reproducability that helps foster new scientific discoveries, namely where rerunning the same analysis on data collected on an equivalent task in another laboratory. In other words, analysis pipelines should ideally be reusable across laboratories. Although it is often overlooked by users (and some developers), care must be taken regarding the license governing use of a given software package in order to maximize its impact. The MNE-Python code is thus provided under the very permissive open source new BSD license. This license allows anybody to reuse and redistribute the code, even in commercial applications.

Neuroimaging is a broad field encompassing static images, such as anatomical MRI as well as dynamic, functional data such as M/EEG or fMRI. The MNE software already relies on some other packages such as FreeSurfer for anatomical MRI processing, or Nibabel for file operations. Our ambition is of course not to make MNE-Python self-contained, dropping any dependency on other software. Indeed the MNE-Python package cannot and does not aim to do everything. MNE has its own scope and seeks to leverage the capabilities of external software in the neuroimaging software ecosystem. Tighter integration with fMRI analysis pipelines could be facilitated by NiPype (Gorgolewski et al., 2011) but is first made possible by adopting standards. That is why all data MNE produces are stored in FIF file format which can be read and written by various software packages written in different languages. The MNE-Python code favors its integration in the scientific Python ecosystem via the use of *NumPy* and *SciPy*, and limits effort duplication and code maintainance burden by pushing to more general purpose software packages any improvement to non M/EEG specific algorithms. For example, improvement of ICA code were contributed back to *Scikit-Learn*, as it could be for signal processing routines in the *scipy.signal* module.

Good science requires not only good hypotheses and theories, creative experimental design, and principled analysis methods, but also well-established data analysis tools and software. The MNE-Python software provides a solid foundation for reproducible scientific discoveries based on M/EEG data. Through the contributions and feedback from a diverse set of M/EEG researchers, it should provide increasing value to the neuroimaging community.

### Conflict of interest statement

The authors declare that the research was conducted in the absence of any commercial or financial relationships that could be construed as a potential conflict of interest.

## Footnotes

^1^http://www.ohloh.net/p/MNE.

## References

[B1] Aine C. Sanfratello L. Ranken D. Best E. MacArthur J. Wallace T. (2012). MEG-SIM: a web portal for testing MEG analysis methods using realistic simulated and empirical data. Neuroinformatics 10, 141–158 10.1007/s12021-011-9132-z 22068921 PMC3543124

[B2] Becker R. A. Cleveland W. S. Shyu M.-J. Kaluzny S. P. (1996). A Tour of Trellis Graphics. Technical Report, Bell Laboratories

[B3] Benjamini Y. Hochberg Y. (1995). Controlling the false discovery rate: a practical and powerful approach to multiple testing. J. R. Stat. Soc. Ser. B 57, 289–300 10.1016/j.neuroimage.2012.07.004

[B4] Buitinck L. Louppe G. Blondel M. Pedregosa F. Mueller A. Grisel O. (2013). API design for machine learning software: experiences from the scikit-learn project. in European Conference on Machine Learning and Principles and Practices of Knowledge Discovery in Databases, Prague

[B5] Carp J. (2012a). On the plurality of (methodological) worlds: estimating the analytic flexibility of fMRI experiments. Front. Neurosci. 6:149 10.3389/fnins.2012.00149 23087605 PMC3468892

[B6] Carp J. (2012b). The secret lives of experiments: methods reporting in the fMRI literature. Neuroimage 63, 289–300 10.1016/j.neuroimage.2012.07.004 22796459

[B7] Dalal S. S. Zumer J. M. Guggisberg A. G. Trumpis M. Wong D. D. E. Sekihara K. (2011). MEG/EEG source reconstruction, statistical evaluation, and visualization with NUTMEG. Comput. Intell. Neurosci. 2011:758973 10.1155/2011/758973 21437174 PMC3061455

[B8] Dale A. Fischl B. Sereno M. (1999). Cortical surface-based analysis i: segmentation and surface reconstruction. Neuroimage 9, 179–194 10.1006/nimg.1998.0395 9931268

[B9] Dale A. Liu A. Fischl B. Buckner R. (2000). Dynamic statistical parametric mapping: combining fMRI and MEG for high-resolution imaging of cortical activity. Neuron 26, 55–67 10.1016/S0896-6273(00)81138-1 10798392

[B10] Delorme A. Makeig S. (2004). EEGLAB: an open source toolbox for analysis of single-trial EEG dynamics including independent component analysis. J. Neurosci. Methods 134, 9–21 10.1016/j.jneumeth.2003.10.009 15102499

[B11] Delorme A. Mullen T. Kothe C. Acar Z. A. Bigdely-Shamlo N. Vankov A. (2011). EEGLAB, SIFT, NFT, BCILAB, and ERICA: new tools for advanced EEG processing. Intell. Neurosci. 2011:130714 10.1155/2011/130714 21687590 PMC3114412

[B12] Desikan R. S. Ségonne F. Fischl B. Quinn B. T. Dickerson B. C. Blacker D. (2006). An automated labeling system for subdividing the human cerebral cortex on MRI scans into gyral based regions of interest. Neuroimage 31, 968–980 10.1016/j.neuroimage.2006.01.021 16530430

[B13] Destrieux C. Fischl B. Dale A. Halgren E. (2010). Automatic parcellation of human cortical gyri and sulci using standard anatomical nomenclature. Neuroimage 53:1 10.1016/j.neuroimage.2010.06.010 20547229 PMC2937159

[B14] Dubois P. (2005). Maintaining correctness in scientific programs. Comput. Sci. Eng. 7, 80–85 10.1109/MCSE.2005.54

[B15] Fischl B. Sereno M. Dale A. (1999). Cortical surface-based analysis ii: inflation, flattening, and a surface-based coordinate system. Neuroimage 9, 195–207 10.1006/nimg.1998.0396 9931269

[B16] Fischl B. Van Der Kouwe A. Destrieux C. Halgren E. Ségonne F. Salat D. H. (2004). Automatically parcellating the human cerebral cortex. Cereb. Cortex 14, 11–22 10.1093/cercor/bhg087 14654453

[B17] Fries P. (2009). Neuronal gamma-band synchronization as a fundamental process in cortical computation. Annu. Rev. Neurosci. 32, 209–224 10.1146/annurev.neuro.051508.135603 19400723

[B18] Gorgolewski K. Burns C. D. Madison C. Clark D. Halchenko Y. O. Waskom M. L. (2011). Nipype: a flexible, lightweight and extensible neuroimaging data processing framework in python. Front. Neuroinform. 5:13 10.3389/fninf.2011.00013 21897815 PMC3159964

[B19] Gramfort A. Keriven R. Clerc M. (2010a). Graph-based variability estimation in single-trial event-related neural responses. IEEE Trans. Biomed. Eng. 57, 1051–1061 10.1109/TBME.2009.2037139 20142163

[B20] Gramfort A. Papadopoulo T. Olivi E. Clerc M. (2010b). OpenMEEG: opensource software for quasistatic bioelectromagnetics. BioMed Eng. OnLine 9:45 10.1186/1475-925X-9-45 20819204 PMC2949879

[B21] Gramfort A. Kowalski M. Hämäläinen M. (2012). Mixed-norm estimates for the M/EEG inverse problem using accelerated gradient methods. Phys. Med. Biol. 57, 1937–1961 10.1088/0031-9155/57/7/1937 22421459 PMC3566429

[B22] Gramfort A. Luessi M. Larson E. Engemann D. Strohmeier D. Brodbeck C. (2013a). MNE software for processing MEG and EEG data. Neuroimage (in press). 10.1016/j.neuroimage.2013.10.027 Available online at: http://www.sciencedirect.com/science/article/pii/S1053811913010501 PMC3930851 24161808

[B23] Gramfort A. Strohmeier D. Haueisen J. Hämäläinen M. Kowalski M. (2013b). Time-frequency mixed-norm estimates: sparse M/EEG imaging with non-stationary source activations. Neuroimage 70, 410–422 10.1016/j.neuroimage.2012.12.051 23291276 PMC3615257

[B24] Gramfort A. Strohmeier D. Haueisen J. Hämäläinen M. Kowalski M. (2011). Functional brain imaging with M/EEG using structured sparsity in time-frequency dictionaries, in Information Processing in Medical Imaging, Vol. 6801 of Lecture Notes in Computer Science, eds Székely G. Hahn H. (Heidelberg; Berlin: Springer), 600–611 10.1007/978-3-642-22092-0_49 21761689

[B25] Granger C. W. J. Hatanaka M. (1964). Spectral Analysis of Economic Time Series. Princeton, NJ: Princeton University Press

[B26] Gross J. Baillet S. Barnes G. Henson R. Hillebrand A. Jensen O. (2013). Good practice for conducting and reporting MEG research. Neuroimage 65, 349–363 10.1016/j.neuroimage.2012.10.001 23046981 PMC3925794

[B27] Gross J. Kujala J. Hamalainen M. Timmermann L. Schnitzler A. Salmelin R. (2001). Dynamic imaging of coherent sources: Studying neural interactions in the human brain. Proc. Natl. Acad. Sci. U.S.A. 98, 694–699 10.1073/pnas.98.2.694 11209067 PMC14650

[B28] Hämäläinen M. Hari R. Ilmoniemi R. Knuutila J. Lounasmaa O. (1993). Magnetoencephalography - Theory, instrumentation, and applications to noninvasive studies of the working human brain. Rev. Modern Phys. 65, 413–497 10.1103/RevModPhys.65.413

[B29] Hämäläinen M. Ilmoniemi R. (1994). Interpreting magnetic fields of the brain: minimum norm estimates. Med. Biol. Eng. Comput. 32, 35–42 10.1007/BF02512476 8182960

[B30] Hunter J. D. (2007). Matplotlib: a 2d graphics environment. Comput. Sci. Eng. 9, 90–95 10.1109/MCSE.2007.55

[B31] Hyvärinen A. Oja E. (2000). Independent component analysis: algorithms and applications. Neural networks 13, 411–430 10.1016/S0893-6080(00)00026-5 10946390

[B32] Klöckner A. Pinto N. Lee Y. Catanzaro B. Ivanov P. Fasih A. (2012). PyCUDA and PyOpenCL: a scripting-based approach to GPU run-time code generation. Parallel Comput. 38, 157–174 10.1016/j.parco.2011.09.001

[B33] Lachaux J.-P. Rodriguez E. Martinerie J. Varela F. J. (1999). Measuring phase synchrony in brain signals. Hum. Brain Mapp. 8, 194–208 10.1002/(SICI)1097-0193(1999)8:4<194::AID-HBM4>3.0.CO;2-C 10619414 PMC6873296

[B34] Larson E. Lee A. K. C. (2013). The cortical dynamics underlying effective switching of auditory spatial attention. Neuroimage 64, 365–370 10.1016/j.neuroimage.2012.09.006 22974974 PMC3508251

[B35] Litvak V. Mattout J. Kiebel S. Phillips C. Henson R. Kilner J. (2011). EEG and MEG data analysis in SPM8. Comput. Intell. Neurosci. 2011:852961 10.1155/2011/852961 21437221 PMC3061292

[B36] Maris E. Oostenveld R. (2007). Nonparametric statistical testing of EEG- and MEG-data. J. Neurosci. Methods 164, 177–190 10.1016/j.jneumeth.2007.03.024 17517438

[B37] Nichols T. E. Holmes A. P. (2002). Nonparametric permutation tests for functional neuroimaging: a primer with examples. Hum. Brain Mapp. 15, 1–25 10.1002/hbm.1058 11747097 PMC6871862

[B38] Nolte G. Bai O. Wheaton L. Mari Z. Vorbach S. Hallett M. (2004). Identifying true brain interaction from eeg data using the imaginary part of coherency. Clin. Neurophysiol. 115, 2292–2307 10.1016/j.clinph.2004.04.029 15351371

[B39] Oostenveld R. Fries P. Maris E. Schoffelen J.-M. (2011). Field Trip: open source software for advanced analysis of MEG, EEG, and invasive electrophysiological data. Comput. Intell. Neurosci. 2011:156869 10.1155/2011/156869 21253357 PMC3021840

[B40] Pantazis D. Nichols T. E. Baillet S. Leahy R. M. (2005). A comparison of random field theory and permutation methods for the statistical analysis of MEG data. Neuroimage 25, 383–394 10.1016/j.neuroimage.2004.09.040 15784416

[B41] Pascual-Marqui R. (2002). Standardized low resolution brain electromagnetic tomography (sLORETA): technical details. Methods Find. Exp. Clin. Pharmacology 24, 5–12 Available online at: http://www.ncbi.nlm.nih.gov/pubmed/12575463 12575463

[B42] Pedregosa F. Varoquaux G. Gramfort A. Michel V. Thirion B. Grisel O. (2011). Scikit-learn: machine learning in Python. J. Mach. Learn. Res. 12, 2825–2830 Available online at: http://jmlr.org/papers/v12/pedregosa11a.html

[B43] Ramachandran P. Varoquaux G. (2010). Mayavi: a package for 3d visualization of scientific data. Comput. Sci. Eng. 13, 40–51 10.1109/MCSE.2011.35

[B44] Ridgway G. R. Litvak V. Flandin G. Friston K. J. Penny W. D. (2012). The problem of low variance voxels in statistical parametric mapping; a new hat avoids a “haircut.” Neuroimage 59, 2131–2141 10.1016/j.neuroimage.2011.10.027 22037420 PMC3361668

[B45] Schelter B. Winterhalder M. Timmer J. (2006). Handbook of Time Series Analysis. Weinheim: Wiley-VCH

[B46] Scherg M. Von Cramon D. (1985). Two bilateral sources of the late AEP as identified by a spatio-temporal dipole model. Electroencephalogr. Clin. Neurophysiol. 62, 32–44 10.1016/0168-5597(85)90033-4 2578376

[B47] Schoffelen J.-M. Gross J. (2009). Source connectivity analysis with MEG and EEG. Hum. Brain Mapp. 30, 1857–1865 10.1002/hbm.20745 19235884 PMC6870611

[B48] Tadel F. Baillet S. Mosher J. C. Pantazis D. Leahy R. M. (2011). Brainstorm: a user-friendly application for MEG/EEG analysis. Comput. Intell. Neurosci. 2011:879716 10.1155/2011/879716 21584256 PMC3090754

[B49] Tallon-Baudry C. Bertrand O. Wienbruch C. Ross B. Pantev C. (1997). Combined EEG and MEG recordings of visual 40 hz responses to illusory triangles in human. Neuroreport NA, 1103–1107 10.1097/00001756-199703240-00008 9175093

[B50] Uusitalo M. Ilmoniemi R. (1997). Signal-space projection method for separating MEG or EEG into components. Med. Biol. Eng. Comput. 35, 135–140 10.1007/BF02534144 9136207

[B51] Van der Walt S. Colbert S. Varoquaux G. (2011). The NumPy array: a structure for efficient numerical computation. Comp. Sci. Eng. 13, 22–30 10.1109/MCSE.2011.37

[B52] Van Essen D. Ugurbil K. Auerbach E. Barch D. Behrens T. Bucholz R. (2012). The human connectome project: A data acquisition perspective. Neuroimage 62, 2222–2231 10.1016/j.neuroimage.2012.02.018 22366334 PMC3606888

[B53] Van Veen B. van Drongelen W. Yuchtman M. Suzuki A. (1997). Localization of brain electrical activity via linearly constrained minimum variance spatial filtering. IEEE Trans. Biomed. Eng. 44, 867–880 10.1109/10.623056 9282479

[B54] Wang J.-Z. Williamson S. J. Kaufman L. (1992). Magnetic source images determined by a lead-field analysis: the unique minimum-norm least-squares estimation. IEEE Trans. Biomed. Eng. 39, 665–675 10.1109/10.142641 1516933

[B55] Wipf D. Nagarajan S. (2009). A unified Bayesian framework for MEG/EEG source imaging. Neuroimage 44, 947–966 10.1016/j.neuroimage.2008.02.059 18602278 PMC4096355

[B56] Wolters C. H. Köstler H. Möller C. Härdtlein J. Grasedyck L. Hackbusch W. (2007). Numerical mathematics of the subtraction method for the modeling of a current dipole in EEG source reconstruction using finite element head models. SIAM J. Sci. Comput. 30, 24–45 10.1137/060659053

